# Perspectives on Participation in a Feasibility Study on Exercise-Based Cardiac Telerehabilitation After Transcatheter Aortic Valve Implantation: Qualitative Interview Study Among Patients and Health Professionals

**DOI:** 10.2196/35365

**Published:** 2022-06-20

**Authors:** Charlotte Brun Thorup, Anne Villadsen, Jan Jesper Andreasen, Jens Aarøe, Jane Andreasen, Barbara Cristina Brocki

**Affiliations:** 1 Department of Cardiothoracic Surgery Aalborg University Hospital Aalborg Denmark; 2 Department of Cardiology Aalborg University Hospital Aalborg Denmark; 3 Department of Sociology and Social Work Aalborg University Aalborg Denmark; 4 Clinical Institute Aalborg University Aalborg Denmark; 5 Department of Physiotherapy and Occupational Therapy Aalborg University Hospital Aalborg Denmark; 6 Public Health and Epidemiology Group Department of Health Science and Technology Aalborg University Aalborg Denmark

**Keywords:** transcatheter aortic valve implantation, aortic valve, implant, TAVI, telerehabilitation, rehabilitation, aortic stenosis, patients’ perspective, older people, elder, aged, geriatric, gerontology, patient experience, user experience, health professional experience, physician experience, telehealth, older adult, telemedicine, cardiac, cardiology, heart, perspective, home-based, exercise, activity tracker, physical activity, mHealth, mobile health, fitness

## Abstract

**Background:**

Aortic valve stenosis affects approximately half of people aged ≥85 years, and the recommended surgical treatment for older patients is transcatheter aortic valve implantation (TAVI). Despite strong evidence for its advantages, low attendance rate in cardiac rehabilitation is observed among patients after TAVI. Cardiac telerehabilitation (CTR) has proven comparable with center-based rehabilitation; however, no study has investigated CTR targeting patients after TAVI. On the basis of participatory design, an exercise-based CTR program (TeleTAVI) was developed, which included a web-based session with a cardiac nurse, a tablet containing an informative website, an activity tracker, and supervised home-based exercise sessions that follow the national recommendations for cardiac rehabilitation.

**Objective:**

This study aims to explore patients’ and health professionals’ experiences with using health technologies and participating in the exercise-based CTR program, TeleTAVI.

**Methods:**

This study is a part of a feasibility study and will only report patients’ and health professionals’ experiences of being a part of TeleTAVI. A total of 11 qualitative interviews were conducted using a semistructured interview guide (n=7, 64% patients and n=4, 36% health professionals). Patient interviews were conducted after 8 weeks of participation in TeleTAVI, and interviews with health professionals were conducted after the end of the program. The analysis was conducted as inductive content analysis to create a condensed meaning presented as themes.

**Results:**

Reticence toward using the website was evident with reduced curiosity to explore it, and reduced benefit from using the activity tracker was observed, as the patients’ technical competencies were challenged. This was also found when using the tablet for web-based training sessions, leading to patients feeling worried before the training, as they anticipated technical problems. Disadvantages of the TeleTAVI program were technical problems and inability to use hands-on guidance with the patients. However, both physiotherapists and patients reported a feeling of improvement in patients’ physical fitness. The home training created a feeling of safety, supported adherence, and made individualization possible, which the patients valued. A good relationship and continuity in the contact with health professionals seemed very important for the patients and affected their positive attitude toward the program.

**Conclusions:**

The home-based nature of the TeleTAVI program seems to provide the opportunity to support individualization, autonomy, independence, and adherence to physical training in addition to improvement in physical capability in older patients. Despite technological challenges, basing the relationship between the health professionals and patients on continuity may be beneficial for patients. Prehabilitation may also be considered, as it may create familiarity toward technology and adherence to the training.

## Introduction

### Background

Aortic valve stenosis (AS) has been reported in approximately 8% of octogenarians [1,2]. Living with AS is associated with risk of morbidity and mortality, and the main symptoms related to this disease are weakness, dizziness, syncope, dyspnea, and chest pain when performing daily activities. Decreased independence and quality of life may accompany the symptoms [3]. Ultimately, untreated AS may lead to heart failure and sudden cardiac death [3].

Transcatheter aortic valve implantation (TAVI) is an alternative to surgical aortic valve replacement or medical treatment in patients with symptomatic severe AS. Compared with surgical aortic valve replacement, TAVI is less invasive and is recommended in older patients (aged ≥75 years) or patients at high surgical risk [2,3]. The number of TAVI surgeries is expected to rise over the coming years owing to demographic developments worldwide and the procedure’s positive short-term and long-term results [2,4,5].

Cardiac rehabilitation (CR) is recommended after surgery as it improves morbidity, functional capacity, and quality of life [6-8]. Participation in CR after TAVI may be of particular importance because sedentary behavior in this population is related to high risk of mortality and functional decline 1 year after the procedure [9]. International guidelines recommend a multidisciplinary CR approach that includes medical and lifestyle risk factor management, cardioprotective therapies, psychosocial management, exercise training, and health behavior change education to improve functional capacity, recovery, psychosocial well-being, and health-related quality of life of patients with cardiac diseases through risk factor modification [10].

In Denmark, participation in CR after TAVI is low, as <20% of patients are referred to and participate in CR [11]. Old age, lack of availability, and individualized rehabilitation seem to reduce the willingness to participate in CR [10,11].

The use of information and communications technologies (ICTs) in home-based CR, termed as cardiac telerehabilitation (CTR) [12,13], has proved to be comparable with center-based and hospital-based CR programs for mortality, cardiovascular events, cholesterol, blood pressure, BMI, cost-effectiveness, and exercise capacity [14-17].

In addition, CTR may provide an option for patients whose rehabilitation needs are not met by existing services, thus improving attendance rates and adherence, as it is performed in the patients’ natural environment and may be incorporated into their daily home routine [17,18].

Supporting older adults in performing exercise at home via a web-based consultation application for a tablet computer was found to be usable [19,20]. In addition, it is expected that different ICTs may be used advantageously to collect and transfer data from the patient to a digital platform or a personal health record in addition to web-based consultation devices for training and communication. However, we have not found any studies that focus on CTR that are based on physical activity and target patients after TAVI surgery. Whether CTR in this population may improve patients’ physical functioning, recovery, psychosocial well-being, and quality of life is unknown. The use of ICTs to improve enrollment and adherence to rehabilitation and support medical and lifestyle risk factor management after TAVI has not been investigated. In addition, it is unknown how patients and health professionals experience participation in CTR. Thus, this perspective requires further investigation to create tailored CTR programs for this target group. On the basis of a participatory design [21], we developed a 12-week digital CTR program, named TeleTAVI, which is ready to be tested in a feasibility study.

### Aim

This study was a part of the feasibility study and will only report on the qualitative findings; therefore, the aim was to explore patients’ and health professionals’ experiences with using health technologies and participating in the exercise-based CTR program, TeleTAVI.

## Methods

### Design

This study reports the qualitative findings from the feasibility study and follows the Consolidated Criteria for Reporting Qualitative Research guideline [22].

### Ethics Approval

Owing to the qualitative nature of the study, the Regional Ethics Committee stated that no approval was required. According to the Helsinki Declaration, oral and written information was provided and informed written consent was obtained from all patients and health professionals. The study protocol was approved by the head of the department and registered by the hospital (ID 2020-054).

### Recruitment

Patients were recruited from the Department of Cardiology, Aalborg University Hospital, Denmark, between March 8, 2021, and May 25, 2021. The TAVI surgery was performed under conscious sedation, and the patients returned to the ward on the same evening or the next morning and were discharged within 2 to 3 days after the surgery.

### Inclusion and Exclusion Criteria

All participants in the qualitative study were the patients and health professionals participating in the feasibility study of TeleTAVI. Inclusion criteria for the patients in the feasibility study were being adults (aged ≥18 years) who underwent a TAVI surgery and could read and understand Danish. The patients in the present cohort were primarily older people with high-risk symptomatic AS. Patients with physical deficits adversely influencing physical performance, as measured by the 6-minute walk test [23], and patients with decreased cognitive functioning, as assessed by the Mini Mental Scale evaluation, were excluded [24]. We also excluded patients with no internet access or low data coverage at their home address. Eligible patients were approached for inclusion on the day before surgery. All patients with cardiac diseases discharged from a Danish hospital can participate in municipality-based CR after the first 8 weeks. This was not an exclusion criterion, but none chose to participate in CR parallel to web-based training. Inclusion criteria for health professionals were having experiences of care, treatment, and rehabilitation targeting older patients with cardiac diseases receiving TAVI.

### Telerehabilitation Technologies and Intervention

TeleTAVI was created as a 12-week digital CTR program that included supervised home-based video training, a web-based session with a cardiac nurse specialist, an informative website, and an activity tracker (Textbox 1) to be used during 8 of the 12 weeks.

**Technologies used in the TeleTAVI program.**

**Tablet (Apple iPad; Wi-Fi; 10.2 inches; 4G)**

- Outlook mail program (Microsoft 365 Office) for web-based exercise training and communication with project personnel via a videoconferencing system, *Videosamtale application*, installed in the unit. The system allowed the physiotherapist to see all the patients on the same screen and communicate with the patients. In addition, patients can see and hear the physiotherapist at the hospital, see themselves and other patients on their screen, and hear all conversations.
- Access to the project´s website
- Visual access to a personal record with information and graphs on uploaded data on daily steps

**Website (Multimedia Appendix 1) hosted by the North Jutland region and installed in the tablet**

- Text information on issues regarding treatment, lifestyle, and medicine
- Videos with training programs
- Videos with patients’ experiences and information from health professionals

**Activity tracker worn on the wrist during daytime (Beurer Activity Sensor AS97; with the use of Beurer AS 97, it was possible to connect and store data on the information technology system used at the hospital, thus complying with the General Data Protection Regulations Compliance Guidelines in Europe)**

- Tracks daily step counts and heart rate during the supervised training sessions.
- Patients were provided an alternative to either upload data on number of daily steps to the personal record or register their daily steps on a personal diary at the end of each day.

**Booklet in paper form**

- Schedule for home visits, web-based training, and self-training and for charging the iPad and the activity tracker
- User manuals for the tablet, website, activity tracker, and Outlook
- Description of home exercises, also illustrated by pictures

**Training equipment used by patients during web-based training sessions**

- Step bench, training mat, elastic exercise band, and dumbbells (1, 2, and 3 kg)

A display on the activity tracker showed the number of daily steps taken, and through uploads, the steps were also visible in the patients’ personal records on the tablet. The activity tracker also had a heart monitor to determine the intensity of the training. In addition, the program included a booklet containing schedules for home visits, web-based training, and self-training; user manuals for the tablet, website, activity tracker, and Outlook mail program; and a description of home exercises illustrated by pictures and written instructions. The website included videos of patients and relatives presenting experiences with TAVI treatment in addition to video-based training programs to be used for unsupervised supplementary training during the first 8 weeks and after, if the patients have their own tablet or computer at home (Multimedia Appendix 1). The patients could choose to maintain the activity tracker for the entire 12-week study period.

The technologies used in TeleTAVI were delivered to the patients in their homes 1 week after hospital discharge. Before hospital discharge, the patients were instructed on a short strength training program for home exercising to be used until the telerehabilitation technologies were delivered to the patient.

To ensure safety during the sessions, the patients were introduced to the exercises and technology by the last author (BCB) during the first home visit. If needed, additional technical support was provided through home visits or telephone calls. The home exercise training was individualized, based on the physical functioning as assessed before surgery and during the first home visit by patients trying the different exercises and equipment to be used in the web-based training sessions. Individualized goals for training were set and followed the national recommendations for CR, with a combination of aerobic and strength training twice weekly, with each session lasting from 30 to 60 minutes [25]. In addition, patients were instructed to take a 30-minute walk daily at moderate intensity.

**Figure 1:**
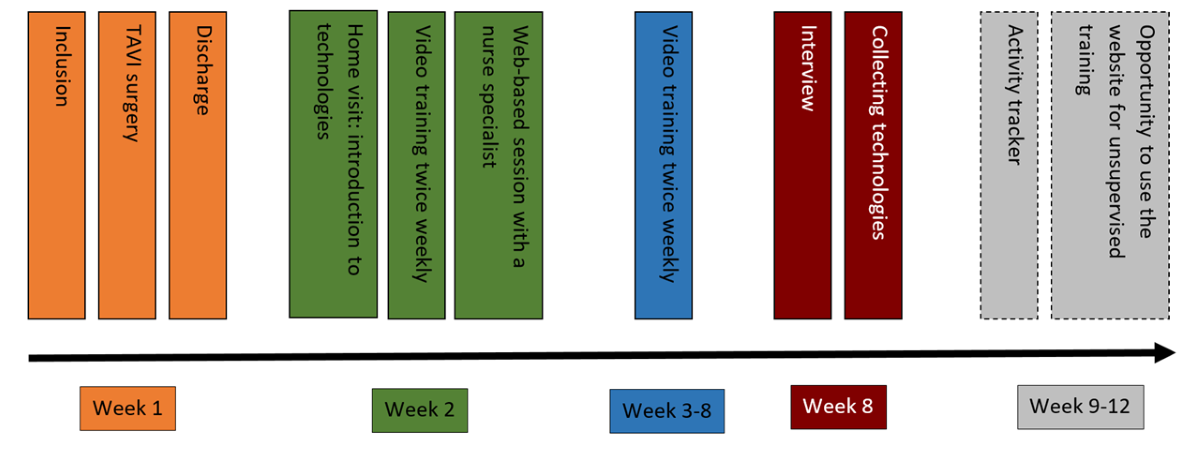
Timeline for TeleTAVI. TAVI: transcatheter aortic valve implantation.

The TeleTAVI program consisted of web-based physiotherapist-supervised exercise training in groups, twice weekly via a tablet; an activity tracker (Textbox 1; Figure 1) that collected the daily number of steps and was able to monitor heart rate; a web-based session with a cardiac nurse specialist; a textbook; and an informative website (Multimedia Appendix 1).

### Qualitative Data Collection and Analyses

The rehabilitation period was 12 weeks, and the duration of the supervised web-based training was 8 weeks, starting in the first week after surgery. After the initial 8 weeks, patients were instructed to continue to exercise unsupervised, and the interviews were conducted at the same time. This time was chosen to gain comprehensive insight into the web-based rehabilitation period without any recall bias.

Individual interviews were conducted with all the patients who had completed the intervention. Furthermore, individual or group interviews were conducted with the involved health professionals. All interviews were based on a semistructured interview guide [26], covering issues such as patients’ experiences with video training, web-based session with a nurse, usability of technology and website, patient support, and issues that the patients felt were important. For health professionals, the focus of the interviews was on their experiences with web-based training and communication. CT (first author) and BB conducted interviews at the patients’ homes, and if present, relatives were encouraged to participate. For the health professionals, the interviews were conducted at the hospital after a minimum of 8 weeks of experience with TeleTAVI for each health professional. These were conducted either as group interviews (physiotherapists) or individual interviews (nurses) for practical reasons. Both interviewers had previous experiences with conducting qualitative interviews. The analysis was conducted as inductive content analysis to create a condensed meaning presented as themes [27]. The recorded interviews were transcribed verbatim and read several times to familiarize with the text and obtain an overall sense of the data. Then, the data were coded for manifest and latent content to identify themes that elaborated the underlying meaning using the coding system, NVivo (version 8; QSR International) [28]. The analyses and interpretations were primarily performed by CBT, AV (second author), and BCB based on their agreement that essential themes were reached [29]; then, the findings were discussed in the research group.

## Results

### Overview

During the study period, 41 patients were referred to TAVI and screened for eligibility. Of these 41 patients, 13 (32%) patients declined to participate, and 13 (32%) patients lived in a place with no internet connection or poor data coverage, thus failing to meet the inclusion criteria. Of the 37% (15/41) of the patients enrolled in the study, 47% (7/15) withdrew after the surgery owing to tiredness (2/7, 29%), non–cardiac-related hospital readmission (2/7, 29%), fluctuating health (1/7, 14%), and regretting participation when the health technologies were introduced during the home visit (2/7, 29%). Furthermore, 7% (1/15) of the patients died before hospital discharge. All patients (7/7, 100%) who completed the TeleTAVI program were interviewed (Table 1). In addition, 2 nurses and 2 physiotherapists (Table 2) who participated in the telerehabilitation program were interviewed. The interviews lasted between 35 and 50 minutes for patients, between 15 and 25 minutes for nurses, and 55 minutes for the group with the physiotherapists.

The patients were aged between 74 and 90 years and had different experience levels in using a tablet or computer. Patients with no experience were able to receive guidance from their relatives, making it possible to complete the telerehabilitation program. In addition, the patients had different physical capability levels after the TAVI procedure, as some felt limited in performing sport and exercise. Finally, some of the patients had comorbidities that may have affected their physical capability, and 57% (4/7) of them had Tilburg frailty score ≥5, indicating frailty in these patients. Regarding education level, 14% (1/7) of the patients had primary school education, 71% (5/7) had vocational education, and 14% (1/7) had higher education (Table 1). The health professionals were aged between 25 and 42 years and had work experience ranging from 2 to 13 years (Table 2).

The analysis and interpretation of the patients’ and health professionals’ interviews resulted in 3 themes and 9 subthemes (Textbox 2).

The interviews revealed that patients and health professionals experienced diverse technological issues. All patients expressed interest to participate in the TeleTAVI rehabilitation program, but none of them were inspired or motivated by the health technologies used. Despite being challenged when using the technologies, they were all optimistic about participation.

**Table 1:** Characteristics of the interviewed patients (N=7).

ID	Sex	Age^a^ (years)	IT experience	Living alone	Education level	Tilburg frailty score^b,c^	Comorbidities	Limitation in doing sport or exercise
P^d^1	Male	85	PC	No	Vocational education^e^	2	AF^f^	Low
P2	Female	90	Tablet	No	Higher education	9	IHD^g^ and AH^h^	Moderate
P3	Female	84	Tablet	Yes	Primary school	2	AH	None
P4	Male	87	None	Yes	Vocational education	8	AF and IHD	High
P5	Male	82	Tablet and PC (spouse)	No	Vocational education	6	IHD	None
P6	Female	74	Tablet	Yes	Vocational education	4	AH	Moderate
P7	Female	81	Tablet and PC (spouse)	No	Vocational education	9	None	Moderate

^a^Median: 84 (range 74-90) years.

^b^Median: 6 (range 2-9).

^c^Score ≥5 points is considered as frailty when using the Tilburg frailty score [30,31].

^d^P: patient.

^e^Primary school and 2 to 5 years of vocational education.

^f^AF: atrial fibrillation.

^g^IHD: ischemic heart disease.

^h^AH: arterial hypertension.

**Table 2:** Characteristics of the health professionals (N=4).

ID	Sex	Age^a^ (years)	Profession	Work experience^b^ (years)
HP^c^1	Female	33	Nurse	5
HP2	Female	42	Nurse	13
HP3	Male	34	Physiotherapist	3
HP4	Male	25	Physiotherapist	2

^a^Range: 25-42 years.

^b^Range: 2-13 years.

^c^HP: health professional.

**Themes and subthemes derived from the content analysis.**

**Technological challenges**

- Reticence toward using the website
- High interest and low competence regarding the activity tracker
- Anticipating technical problems led to worries beforehand.

**Advantages of home-based training**

- Individualization despite lack of hands-on guidance and technical problems
- Home-based training reduces transportation time and may support adherence.
- Proper exercise was done.

**The importance of establishing a relationship**

- Web-based training does not support relatedness between patients.
- First visit clears the way and creates a base for continuity.
- No recall of the web-based session with the nurse.

### Technological Challenges

#### Reticence Toward Using the Website

Most of the patients (6/7, 86%) did not visit the website. Some were not curious about the content, whereas others were not familiar with the technologies and were afraid of making errors, such as deleting elements unintentionally. A participant described the following:

Actually, we haven’t [visited the website]. We were afraid of pressing a key that would delete some of the content.

P1

The participants’ concern about making errors limited the use of the website, and therefore, they did not fully benefit from the knowledge and coping elements provided on the website. Therefore, the patients’ overall benefit from the website may be questioned.

Other reasons for not visiting the website were reluctance toward being involved in other patients’ experiences of the disease:

I’ve always felt like this...I can’t stand to hear about disease, about faults in the heart.

P2

This finding shows that some elements on the website may have unintentional consequences by triggering additional anxiety and fear when confronted with other patients’ experiences of living with heart disease.

Furthermore, few patients (2/7, 29%) watched the training videos, and only 14% (1/7) of the patients performed additional workouts. They argued that training twice a week with the physiotherapist felt sufficient.

Overall, the participants had neither the skills nor the curiosity to explore the content of the tablet, meaning that they did not make full use of the opportunities given. Others did not want to be confronted with other patients’ life stories.

#### High Interest and Low Competence Regarding the Activity Tracker

All patients (7/7, 100%) found the activity tracker exciting to use, as they could keep track of how many steps they had walked, and the tracker made it possible to compare steps from day to day, which facilitated motivation. All but 1 patient (6/7, 86%) preferred to write down the daily steps in a diary, as it was very difficult to upload daily steps via Bluetooth to their health record. In addition, some patients missed the information that the tracker had to be recharged once a week, leading to additionally missing electronically uploaded step data. Furthermore, the monitoring of heart rate during exercise was difficult for the patients to learn, meaning that the intended use of heart rate to determine exercise intensity was lost:

Well, you think more about it [improvements]...and you are excitedly waiting...Oh, today you have walked this much.

P7

That’s the only thing I have used it for, measuring steps. I have not measured heart rate...well, I think, I have learned to measure steps, lets stick to that. Because I am not fond of learning new things...I would rather avoid it.

P2

Although the activity tracker motivated walking activities, the participants were challenged technically. Thus, the potential motivational factor for the patients to follow their increase or decrease in daily steps walked was hampered, and the health professional’s opportunity to continuously follow the patients’ walking activity was lost for most of the patients (5/7, 71%).

#### Anticipating Technical Problems Led to Worries Beforehand

Some patients described preparation for sessions as slightly compulsive and held themselves in readiness twice a week, worrying that technical problems would occur, such as failing to log in or malfunctioning sound and vision in the tablet. The patients described the technical problems during training as problems with hearing the physiotherapist clearly, stuttering web-based transmission, and feeling interrupted by other patients:

It was scaring, I was worried for the web-based sessions. In good time I was sitting in front of the tablet, wondering whether it would work this time or not. Sometimes I couldn’t hear or see anything, or they could not hear me.

P2

Thus, the patients reported that waiting for the web-based sessions twice a week felt slightly involuntary and created worries that something would go wrong.

### Advantages of Home-Based Training

#### Individualization Despite the Lack of Hands-on Guidance and Technical Problems

The physiotherapists reported technical problems during the web-based training sessions, such as problems with logging in and handling the sound and picture settings. Delays in starting the session and interruptions during training were frequent. This led to 2 physiotherapists being present during sessions, one managing technological problems and the other conducting the training sessions:

Sometimes, they needed a lot of guidance because...then they switched the camera on in the wrong direction, or the screen did “freeze” or, they just happened to turn it off. You had to guide the patients through all this, and when the training had begun, another physiotherapist guided the patient through technical problems.

HP3

The physiotherapists questioned the quality of the exercises performed, as the web-based training made it difficult for them to guide the patients in the usual hands-on way. In addition, intermittent poor internet connections, stuttering images, and very small images made it difficult for the physiotherapists to see whether the exercises were performed correctly and properly by the patients.

The physiotherapists observed great diversity in the patients’ physical and respiratory fitness, thus spending more time on patients who were most challenged. However, they strived to achieve individualization, to support all patients in gaining improvements in fitness level despite individual capabilities. For the physiotherapists, the individualization seemed double-sided as it was time-consuming; however, it created a possibility for all patients to improve their level of physical capability. On the basis of these experiences, the physiotherapists suggested that in future interventions, patients should be divided into groups based on their level of physical capacity.

Similarly, the patients valued that the training was individualized and appreciated being guided in the correct execution of the exercises:

And when there is something you can’t do, then he [the physiotherapist] always corrects you in a good way.

P5

This meant that the physiotherapists had to accept that the quality of the training might be reduced, as hands-on guidance was not possible together with the patients’ limited technical skills and slow internet connections. However, the patients expressed that the opportunity for individualization supported improvement.

#### Home-Based Training Reduces Transportation Time and May Support Adherence

Most of the patients (5/7, 71%) increased their web-based technical competencies, whereas others needed help throughout the study period. Despite the web-based technical problems, both patients and physiotherapists provided several positive feedbacks about the home-based web-based training. All patients (7/7, 100%) appreciated being able to exercise in their homes because it felt safe, and they avoided transportation to a rehabilitation center, which was one of the main reasons for participating in TeleTAVI for all patients:

I did benefit from participation, also in a physical way, by experiencing that nothing fatal would happen during exercise...I did manage it without collapsing halfway through.

P1

I mean, it’s been easy because you were at home, and you didn’t have to drive for it...and the time [was used properly].

P7

In addition, some patients felt inspired to continue web-based training after 8 weeks of participation. This was consistent with thoughts expressed by physiotherapists, who stated that home-based exercise might motivate patients to continue training after the project has ended because the patients were taught in their homes and felt safe while performing the exercises there:

Well, we can just keep on doing the exercises...we will continue to do that.

P5

The advantage is that it’s easily transferable for them [the patients] afterward.

HP3

Thus, avoiding the costs and time spent on transportation and the expectation that home-based training may support adherence to future training activity were positive aspects of participation.

#### Proper Exercise Was Performed

All but 1 of the patients (6/7, 86%) felt physical improvement at the time of the interviews. Whether improvements were mainly caused by the TAVI surgery, telerehabilitation, or most likely, a mixture of both cannot be determined in this study.

Despite considerations about own physical ability, approximately all patients felt that they had performed a proper and satisfying exercise for the whole body, leading to a natural feeling of tiredness after the sessions. Some participants became more energized and apprehended that less dyspnea made them capable of exercising at a high level:

Well, I can feel that it’s good for my body. It’s like, I become livelier and light. And I think that you gain energy when you do exercise...when I am finished with workout, my hair is wet, and my clothes needs washing.

P6

I have realized that exercise helps you, and now...as I am less dyspnea, more comfortable and my feet got smaller. I can actually be more active and not just “dragging around.”

P4

Being under surveillance and encouraged to challenge one’s comfort zone during exercise seemed to increase the benefit of training. The patients’ old age was kept in mind while considering their expectations of improvement, realizing that there must be a limit on how much improvement to expect. In contrast, a patient expressed that exercise may “stop the clock” and add extra time to their life:

I think, when you are forced to do more than you actually can, then it pays back...and I do more during training than I would have done on my own...You have to remember that my body is 90 years of age; it is limited how much better I can become.

P2

You got sweatier exercising with the physiotherapist than when you exercise on your own... [when becoming older] training might stop the clock.

P1

On the basis of the physiotherapists’ observation of patient improvements, they agreed that the web-based sessions fulfilled the goals for exercise training, such as improvements in fitness level and muscle strength, and increased the feeling of being safe during training:

It succeeded because their general functional level and increased age do “set them back” [physically], meaning that just small efforts provide minor improvements; even though the patients don’t do the exercise correctly, there might still be physical progress.

HP3

Thus, patients and physiotherapists felt that exercise improved physical fitness, and both surveillance and encouraging pressures seemed to support the patients’ feeling of exercising safely.

### The Importance of Establishing a Relationship

#### Web-Based Training Does Not Support Relatedness Between Patients

The physiotherapists expressed worries that the TeleTAVI would reduce patients’ opportunity of being socially involved with each other. For the patients, being socially involved with others seemed less important and did not influence their willingness to participate in the program. Being able to see others during training did not have any importance for most patients (6/7, 86%), as they were just considered as “images on the screen” and not as living persons. Others were only noticed if they disturbed the sessions. When relatives participated in the exercise with the patients, they performed the exercise outside the reach of the camera:

The most annoying about the tablet, is that you are forced to look at the others while training. The others don’t matter to me because I don’t look at them as persons [...]. And one, she was talking so loudly...I couldn’t understand what the turmoil was about [...]. I was very close to just turning the s*** off; I couldn’t deal with all that commotion.

P6

As such, being socially related to other patients was not considered as an important issue by the patients.

#### First Visit Clears the Way and Creates a Base for Continuity

All patients (7/7, 100%) had the training equipment and tablets delivered at home. Being introduced to the program and having the opportunity to try both the technologies and training equipment was beneficial in preparing the patients for the following web-based sessions. Some patients felt excited, whereas others expressed that meeting the physiotherapists was nice and helped them in performing the exercise safely and properly:

It was really nice [having the technology and equipment brought to the home], we had a really good talk, and I was happy to be shown how to do the exercises properly.

P1

The relationship established between BCB and the patients during the first home visit and through additional visits became important, and they felt grateful. In particular, physiotherapists’ ability to be attentive toward each individual patient during the web-based exercise sessions made the patients feel that they could participate on equal terms. Continuity in the relationship with the health professionals was expressed as very important. The patients felt acknowledged as individuals, and thus safe.

Thus, it was evident that the first home visit affected the relationship with the patients positively and supported a good start within the program. In addition, seeing the same person at the first home visit and several times during the rehabilitation period positively influenced the patients’ attitudes toward the program.

#### No Recall of the Web-Based Session With the Nurse

During the nurse session, 1 week after discharge, both nurses experienced difficulties with the video call, meaning that some of the sessions were performed as telephone calls. Both nurses expressed that video calls had many advantages, such as being able to see the patients’ physical condition and obtain a sense of their mental and psychological well-being. The video call provided security and continuity for the patients, as they may have met the nurse during hospitalization:

They were happy about it [the web-based session]. I think for them it felt comforting that someone did follow up on them about these things [health and well-being after discharge].

HP2

Most patients (6/7, 86%) did not remember talking to a nurse, and if they remembered, they had little or no recollection of what was discussed:

A nurse? ...I think it was on the phone...no, wait, it might have been one from the healthcare center, sorry, I can’t tell. And I can’t remember what we talked about.

P3

Thus, despite good experiences from the nurses’ point of view, none of the patients could recall the session’s content, and most of them (6/7, 86%) did not remember the session at all. An explanation may be that this group of older people have numerous contacts with health professionals, either from the hospital or the primary sector, making it difficult to discriminate between the health professionals. However, it seems to underpin the importance of supporting continuity in the contact with the patients and to question the timing and content of the call.

## Discussion

### Principal Findings

This study aimed to explore patients’ and health professionals’ experiences with using health technologies and being part of the exercise-based CTR program, TeleTAVI.

In summary, the interviews indicated that patients were slightly reticent toward using the website, they had neither the skills nor the curiosity to explore the tablet, and some avoided other patients’ life stories. The activity tracker held much interest from the patients, but they did not gain full benefit from it as their technical competencies were low. This was also a problem when using the tablet for web-based training sessions, leading to patients feeling worried before the training, as they anticipated technical problems. The physiotherapists expressed that the disadvantages were technical problems and not being able to use hands-on guidance when the patients’ exercises were performed inappropriately or incorrectly. In contrast, they expressed that improvements were observed in patients’ physical fitness and that training at home created a feeling of safety and supported adherence to training. In addition, they avoided transportation to a training center. Despite the low quality of pictures and, sometimes, the internet, the physiotherapists made individualization possible. Individualization was valued by the patients, and the they felt that they exercised properly and felt improvement during the rehabilitation period. Good relationships and continuity in contact were extremely important for the patients and affected their attitude toward the program; when having only one contact, as the nurses did, the recall of the session was hampered. None of the patients (0/7, 0%) expressed a wish to be socially related to other participants during the program.

### Comparison With Previous Work

#### Technological Challenges

Technological challenges were evident when talking to the health professionals. These findings are consistent with those of a review by Fischer et al [32] on the acceptance and use of health technology in older people. They concluded that older people face different challenges, such as limited familiarity with technology, reticence about asking for assistance, mistrust, and concerns about privacy when using technology [32]. This was similar to the patients in this study who were reticent toward using the technology and had neither the skills nor the curiosity regarding the use of the tablet and website. In addition, they were afraid of deleting something from the tablet, which refrained them from exploring possibilities it.

Older patients’ preferences and attitudes toward digital technology have been investigated by Terp et al [33], who concluded that lack of knowledge, user competence, and interests were the main barriers to older people’s use of technology. These findings are consistent with the results of this study, in which the patients did not use the full potential of the tablet, activity tracker, or heart monitor and none of them participated owing to interest in using health technologies. This underpins that devices targeting older people should be easy to use and recharge, with automatic uploading of data.

Another explanation for the low use of technology may be related to their level of education. Hargittai et al [34] studied internet skills among citizens aged ≥60 years and concluded that high income and high level of education equals high level of internet skills. This contributes to the explanation of why patients in this study experienced problems in using the tablet and activity tracker. It is interesting that not being interested in or not having experience with technology did not discourage them from participation; in contrast, they were glad to do so, which indicates that they were not afraid of trying new and unknown technology. This is consistent with Fischer et al [32], who concluded that older people generally have a positive attitude toward using health technologies at their homes, if they believe that it will help the health professionals to preserve their independence and autonomy. In addition, they seem less bothered when technical problems arise; they just wait for problems to be solved [33]. In this study, the patients did not express any annoyance when facing technological issues. They got used to 2 physiotherapists being present during the exercise sessions, one helping with technologies and the other guiding the training, and they did so without questioning this high use of health personnel resources.

Thus, mistrust toward technology and reduced familiarity, competencies, curiosity, and trust in one’s own ability were important factors that influenced the patients’ willingness and possibility of using the technology, resulting in not gaining full benefit from the technology used. In contrast, this did not discourage them from participating in the CTR program in which the training could be conducted in their home environment.

#### Home-Based Training and Establishing a Relationship

The patients were satisfied with avoiding transportation and being able to exercise at home. They felt having performed proper exercise during the web-based sessions and felt improvement in health, physical status, and life in general. However, life expectancy and quality of life may not mean the same across generations. Leeuwen et al [35] found that, for older adults, quality of life is related to autonomy, independence, and ability to handle life circumstances and potential changes that come with increased age. In addition, experiences of one’s own health seem relative as experiences with health depend on circumstances and what a person finds to be reasonable in relation to their age, history, medical condition, and social situation [35]. A study on older men’s perspectives on good and healthy aging reveals that becoming older is balancing having expectations and ambitions for your life and realizing the realities about your physical and social situation. This balance may be handled either by improving your circumstances in life or lowering your ambitions and expectations [36]. They concluded that physical and cognitive health was important to ensure independence and autonomy [36]. In this study, the patients felt improvement, whereas the physiotherapists questioned the quality of exercise; however, it might be argued that life expectancy and quality of life in older people may relate more to maintaining independence and autonomy than an increased physical health level. Thus, their expectations for participating in telerehabilitation may be the improvement of life circumstances, while at the same time, accepting their everyday life capabilities.

Social interaction and connectivity to peer fellows have previously been considered as important in supporting behavior changes, such as increasing physical activity [37,38]. However, it is argued that some commonly used self-regulation intervention techniques that are effective for younger adults may not be effective for older adults [38]. This is consistent with our study, in which none of the patients expressed a wish to be socially related to other participants during the program.

Professor in Sociology, Arthur Frank [39], describes that one way of experiencing and talking about illness is to rely on a restitution narrative that holds the expectation that a sick body will become better. All the patients in TeleTAVI participated owing to expectations of becoming better and recovering, and they trusted that telerehabilitation would support this. Thus, it can be argued that as the patients’ narratives follow this restitution model [39], it is apparent that the patients feel motivated to participate in a rehabilitation program such as TeleTAVI because their main goal is to recover after their TAVI surgery. This could mean that there is great incentive to continue to develop telerehabilitation for older patients in general because, from the patients’ perspectives, there are several strengths to be found in telerehabilitation.

In summary, older people may view life expectancy and quality of life as a balance between life expectations and circumstances regarding age, physical and medical condition, and social situation, and motivation for participation in CTR may originate from a restitution narrative. It can be argued that this group of patients might be the ideal target group for telerehabilitation because their advanced age and decreased activity reduce their willingness to participate in rehabilitation outside their homes and individualized exercise performed in their own homes may increase independence, autonomy, and adherence. Hence, the main challenge for telerehabilitation is to restructure and further develop it to make it more efficient and feasible, which will be discussed in the following section.

### Future Directions

One of the main strengths of telerehabilitation that both the health professionals and the patients emphasized was the possibility to individualize the web-based exercise sessions. Eichler et al [40] argued that individualization of CR after TAVI is important to maintain the patients’ autonomy.

A study by Eichler et al [40] about geriatric versus CR concluded that it may be rewarding to classify the degree of frailty regarding older patients’ technological and physical skills. Dividing patients into different groups would ensure that those who need more support receive it. This was consistent with the suggestions from the physiotherapists in this study.

In addition, individualization and enhanced efficacy may be reached by starting rehabilitation before the TAVI surgery as prehabilitation. Pighi et al [41] investigated the determinants of outcomes after TAVI and found that high frailty before surgery negatively affects the outcome of the surgery, particularly in women. Therefore, Pighi et al [41] suggested the implementation of prehabilitation to support the physical status of patients who are frail and to have better outcome after TAVI [41]. Prehabilitation also holds the opportunity to practice their technological skills before the surgery.

In this study, training at home supported the patients in feeling safe, and it was expected to enhance adherence to exercise after the rehabilitation period. The long-term effects of exercise training after TAVI have been investigated by Pressler et al [42], who argued that it is important to continue exercising to maintain improvements in the long term, but most fail to adhere to exercise [42].

Thus, the feasibility of TeleTAVI in the current form may still be questioned; however, it holds potential for the future. In particular, the home-based nature of the program seems to contain possibilities for supporting individualization, autonomy, independence, and adherence in addition to supporting improvement in physical capability in older patients. Considering that some older patients are digital immigrants and may oppose new technology, prehabilitation can advantageously be implemented to support familiarity toward technologies and execution of exercise.

For the patients, having a good relationship with health professionals seemed important for their attitude toward the program, and continuity in meeting the same health professional more than once added to the positive experience of having a good relationship. According to research by Feo et al [43] and Bridges et al [44], a positive, trusting relationship supports dignity and helps patients to make informed decisions in addition to supporting the delivery of high-quality care. Therefore, it seemed beneficial to support continuity in the interactions between health professionals and patients when conducting exercise-based CTR.

### Strengths and Limitations

This study has some limitations. First, the small number of participants resulted in reduced data saturation; however, rich data were gathered, which allowed for in-depth exploration of patients’ and health professionals’ experiences of participating in TeleTAVI [45]. Second, the interviews were conducted after 8 weeks of web-based training, meaning that it was impossible to gain insight into experiences of long-term adherence to training.

However, this study has several strengths. Despite the low number of participants, the group of patients reflected the population in the following areas: age, comorbidities, and education level [46]. Another strength is that all interviews were conducted in the patients’ private homes to support a relaxed and comfortable atmosphere, to make the patients feel safe, and both interviewers were experienced in conducting semistructured interviews with patients with cardiac diseases. In addition, it is considered a strength that both patients and health professionals involved in the telerehabilitation were interviewed to ensure deep and more comprehensive understanding of the feasibility of the telerehabilitation program.

Finally, another strength of the study is that CBT and AV undertook in-depth reading and analysis to avoid misinterpretation of the interviews, and an ongoing discussion of the analysis and interpretation was conducted with the last author (BCB) to ensure rigor and credibility.

### Conclusions

In conclusion, to the best of our knowledge, this was the first investigation of patients’ and health professionals’ experiences of exercise-based CTR for patients following TAVI surgery.

The feasibility of TeleTAVI in the current form may be questioned. The patients were reticent toward using the website, and they had neither the skills nor the curiosity to explore the tablet. They found the use of the activity tracker interesting, but full benefit was not achieved from it. The patients had low technical competencies, leading to feeling worried before the training, as they anticipated technical problems. The physiotherapists observed improvements in patients’ physical fitness despite technical problems and that home training supported safety, individualization, and adherence. Individualization was valued by the patients, and they experienced physical improvements. Good relationships and continuity in contact with health professionals were extremely important for the patients, and they did not express any wish to be socially related to the other participants during the program.

However, TeleTAVI holds potential for the future. In particular, the home-based nature of the program seems to contain possibilities for supporting individualization, autonomy, independence, and adherence in addition to supporting improvement in physical capability in older patients. In addition, basing the relationship on continuity between health professionals and patients may be beneficial for patients. Considering that some older patients are digital immigrants and may oppose new technology, prehabilitation can advantageously be implemented to support familiarity toward technologies and execution of exercise.

## Appendix

Multimedia Appendix 1 Informative website created for TeleTAVI.

## Abbreviations

AS: aortic valve stenosis
CR: cardiac rehabilitation
CTR: cardiac telerehabilitation
ICT: information and communications technology
TAVI: transcatheter aortic valve implantation
