# Supplementary material for: Perspectives on Participation in a Feasibility Study on Exercise-Based Cardiac Telerehabilitation After Transcatheter Aortic Valve Implantation: Qualitative Interview Study Among Patients and Health Professionals
Source: JMIR Form Res. 2022 Jun 20;6(6):e35365. doi: 10.2196/35365 (PMC9253976; doi:10.2196/35365)
Supplement: Multimedia Appendix 1 [file formative_v6i6e35365_app1.docx]

**Multimedia Appendix 1. Informative website created for TeleTAVI.**


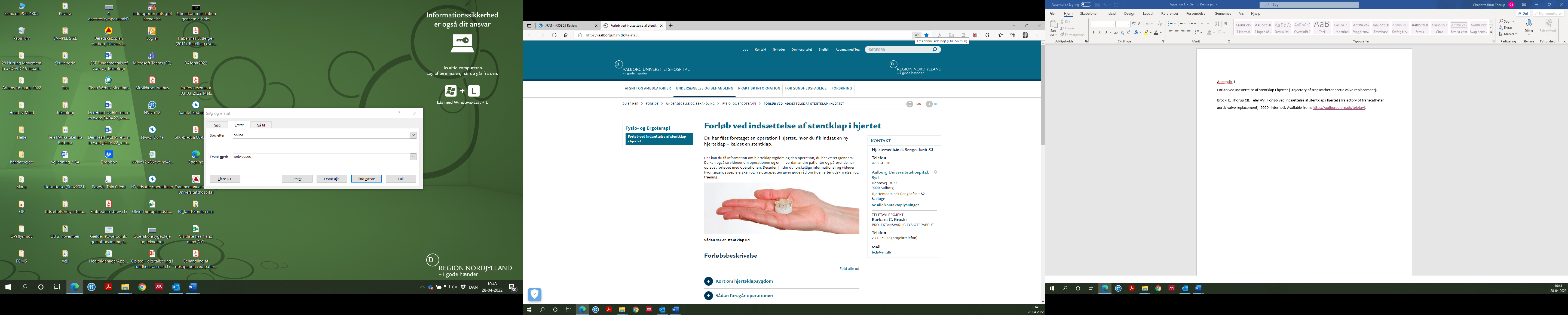


Brocki B, Thorup CB. TeleTAVI. Forløb ved indsættelse af stentklap i hjertet (Trajectory of transcatheter aortic valve replacement); 2020 [Internet]. Available from: <https://aalborguh.rn.dk/teletavi>.
